# Avoidable 30‐day readmissions in patients undergoing vascular surgery

**DOI:** 10.1002/bjs5.50191

**Published:** 2019-08-02

**Authors:** A. Knighton, G. Martin, V. Sounderajah, L. Warren, O. Markiewicz, C. Riga, C. Bicknell

**Affiliations:** ^1^ Department of Surgery and Cancer Imperial College London London UK; ^2^ Imperial Vascular Unit Imperial College Healthcare NHS Trust London UK

## Abstract

**Background:**

Vascular surgery has one of the highest unplanned 30‐day readmission rates of all surgical specialties. The degree to which these may be avoidable and the optimal strategies to reduce their occurrence are unknown. The aim of this study was to identify and classify avoidable 30‐day readmissions in patients undergoing vascular surgery in order to plan targeted interventions to reduce their occurrence, improve outcomes and reduce cost.

**Methods:**

A retrospective analysis of discharges over a 12‐month period from a single tertiary vascular unit was performed. A multidisciplinary panel conducted a manual case‐note review to identify and classify those 30‐day unplanned emergency readmissions deemed avoidable.

**Results:**

An unplanned 30‐day readmission occurred in 72 of 885 admissions (8·1 per cent). These unplanned readmissions were deemed avoidable in 36 (50 per cent) of these 72 patients, and were most frequently due to unresolved medical issues (19 of 36, 53 per cent) and inappropriate admission with the potential for outpatient management (7 of 36, 19 per cent). A smaller number were due to inadequate social care provision (4 of 36, 11 per cent) and the occurrence of other avoidable adverse events (4 of 36, 11 per cent).

**Conclusion:**

Half of all 30‐day readmissions following vascular surgery are potentially avoidable. Multidisciplinary coordination of inpatient care and the transition from hospital to community care after discharge need to be improved.

## Graphical abstract

Fifty per cent of all unplanned 30‐day readmissions in patients undergoing vascular surgery are potentially avoidable. To reduce this burden, there is a need to take a multidisciplinary approach to optimizing medical care, support the transition from hospital to community care effectively, and develop targeted strategies to patients deemed to be at high risk.

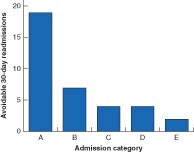

Many readmissions avoidable

## Abstract (es)

**Antecedentes:**

La cirugía vascular tiene una de las tasas más elevadas de reingresos no planificados a los 30 días de todas las especialidades quirúrgicas. Se desconoce hasta qué punto este problema puede ser evitable y las estrategias óptimas para su disminución. El objetivo de este estudio fue identificar y clasificar los reingresos evitables a los 30 días en pacientes sometidos a cirugía vascular para planificar intervenciones dirigidas a su disminución, mejorar los resultados y reducir el coste.

**Métodos:**

Se realizó un análisis retrospectivo de las altas hospitalarias durante un periodo de 12 meses en una unidad vascular terciaria. Un panel multidisciplinario realizó una revisión manual de los casos para identificar y clasificar aquellos reingresos urgentes no planificados a los 30 días que se considerasen evitables.

**Resultados:**

Se registró un reingreso no planificado a los 30 días en 72/885 (8,1%) ingresos. Estos reingresos no planificados fueron considerados evitables en el 50,0% (36/72) y fueron debidos con más frecuencia a cuestiones médicas sin resolver (19/36, 52,8%) y a un ingreso no apropiado con la posibilidad de tratamiento ambulatorio (7/36, 19,4%). En un número menor de casos se debió a una asistencia social inadecuada (4/36, 11,1%) y la aparición de otros eventos adversos evitables (4/36, 11,1%).

**Conclusión:**

La mitad de los reingresos a los 30 días en pacientes vasculares son potencialmente evitables. Tras el alta hospitalaria debe mejorarse la coordinación multidisciplinaria de la atención hospitalaria y la transición desde el hospital a la atención comunitaria.

## Introduction

Healthcare systems around the world are increasingly using hospital readmission rates as a marker of quality and performance^1^, ^2^, ^3^. Readmission rates can be used as a financial instrument to drive improvements in performance. For example, the Centers for Medicare and Medicaid Services Hospital Readmissions Reduction Program in the USA and similar programmes in the National Health Service (NHS) in England penalize hospitals via the reduction or non‐payment of care costs associated with emergency readmissions within 30 days of discharge^4^, ^5^. The aim of these policies is to incentivize hospitals to reduce emergency readmissions, which not only represent a significant resource burden but are also associated with poorer outcomes for patients^6^, ^7^. Multiple factors are likely to contribute towards readmissions, including patient‐specific factors such as socioeconomic status, medical co‐morbidities and disease severity, as well as factors related to the care patients receive in the course of their hospital admission or during the transition from secondary to community care after discharge^7^.

Despite the introduction of financial penalties, emergency readmissions have continued to increase, rising by 22·8 per cent in England between 2012–2013 and 2016–2017^8^. This raises serious questions regarding clinical decision‐making and support for transitions in care after discharge for a large number of patients receiving inpatient treatment. Emergency readmissions not only represent a significant cost burden to the wider healthcare economy (an estimated £2·4 billion (approximately €2·7 billion, exchange rate 21 June 2019) in 2012–2013 in England and $17 billion (€15 billion, exchange rate 21 June 2019) in the USA in 2004)^4^, ^9^, but also constitute a disproportionate proportion of a hospital's inpatient care expenditure relative to the cost of the index admission^10^.

Patients undergoing vascular surgery are typically frail, co‐morbid, and subject to significant and often numerous interventions during their care. This results in a higher incidence of postoperative complications and subsequent readmission after discharge compared with other surgical specialties^11^. In 2017, the National Vascular Registry in the UK reported a 6 per cent unplanned 30‐day readmission rate for patients undergoing elective open abdominal aortic aneurysm (AAA) repair, which rose to 13·4 per cent in patients having emergency lower‐limb revascularization^12^. This is consistent with readmission rates seen elsewhere, including in the USA^13^.

Despite this, a paucity of literature exists regarding avoidable readmissions in surgical specialties. Only one study^14^ has sought to conduct a standard peer‐review process to examine the avoidability of readmissions in a surgical cohort. Although a number of studies^15^, ^16^, ^17^ have identified the extent of ‘unplanned’ readmissions in vascular surgery, none has ascertained the proportion that may be deemed avoidable through a robust and valid process. Identifying those readmissions deemed to be avoidable would support the implementation of targeted strategies to reduce readmission rates to the benefit of patients, hospitals and the wider healthcare economy.

The aim of this study was to identify patients readmitted to hospital within 30 days of discharge after a vascular inpatient admission, and through a process of expert multidisciplinary peer review to identify and classify those readmissions deemed to be avoidable in order to identify processes and high‐risk populations suitable for future targeted interventions.

## Methods

Analysis was performed of a database compiled over a 1‐year interval between 1 January 2016 and 1 January 2017 at a tertiary vascular surgery service in London, UK. All patient episodes, both elective and emergency, with a length of hospital stay (LOS) greater than 24 h were included. Patients were excluded from analysis due to coding errors (such as errors in administrative data, duplicate data or incorrect specialty coding), day‐case admissions with LOS of less than 24 h, patients repatriated to a local acute hospital after surgery, those who died during the index admission or within 30 days of discharge, and patients under 18 years of age. Ethical approval was not required for this quality improvement and audit project in line with the principles outlined in the Declaration of Helsinki.

The electronic health record of all eligible patients was screened manually, and entries leading to a subsequent readmission to any specialty within 30 days were identified. Data were collected for all readmission episodes, including Index of Multiple Deprivation (IMD)^18^, co‐morbidities, Charlson Co‐morbidity Index score^19^ and standard demographic details. For each readmission episode, the time to readmission, route of admission, primary reason for readmission, procedures undergone, and location of subsequent discharge were also recorded. Planned readmissions, defined as those intended at the time of discharge from the index admission, were excluded, as were patients who self‐discharged early from their index admission against medical advice, as these were deemed not to reflect adequately the quality of care received.

An eight‐member multidisciplinary expert review panel was convened consisting of two senior vascular surgeons, three clinical research fellows in transitions of care, one vascular clinical nurse specialist, an advanced vascular nurse practitioner and a senior specialist vascular pharmacist. The panel first classified unplanned readmissions as avoidable or unavoidable in terms of the following definition of an avoidable readmission: ‘a readmission that, on the balance of probability, whether related or unrelated to the index admission or its complication, could have been prevented with optimal care that is judged to be reasonable within the constraints of the health system’. Readmissions were further classified into one of five categories through consensus following in‐depth case review, in line with previously published methods for classifying and describing avoidable readmissions^14^, ^20^: unresolved issue on discharge (category A); inappropriate admission (B); inadequate social support (C); adverse event (D); and other avoidable readmission (E). For the purposes of classification, adverse events were defined according to the established literature^21^: ‘an unintended injury or illness to a patient caused by medical management rather than the underlying condition of the patient’.

### Statistical analysis

Standard descriptive statistical analysis was performed on demographic data, reporting median and range for non‐parametric data. Fisher's exact, Kruskal–Wallis and Mann–Whitney *U* tests were used to compare differences between avoidable and unavoidable readmission cohorts. All analyses were conducted using IBM SPSS® version 24 for Windows® (IBM, Armonk, New York, USA). Figures were created using GraphPad Prism® version 7.00 for Windows® (GraphPad Software, San Diego, California, USA).

## Results

**Figure 1:**
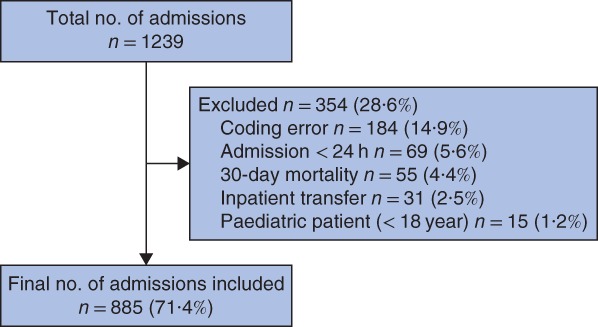
Flow diagram of patients included in the study

Some 1239 discharges were identified during the study period. Of these, 354 (28·6 per cent) were excluded from further analysis (*Fig*. 1). The median age of the 885 discharged patients included in the final analysis was 69·7 (range 19–93) years, 612 (69·2 per cent) were men, and the median index LOS was 9 (range 1–155) days. Some 104 discharged patients (11·8 per cent) were readmitted within 30 days. Of these, 28 patients whose readmission was planned as part of their ongoing care were excluded from further analysis, as were four patients who discharged themselves against medical advice during their index admission.

A total of 72 of the 885 patients (8·1 per cent) had an unplanned 30‐day readmission. Demographic details, co‐morbidities and surgical procedures performed are shown in *Table* 1. Patients were co‐morbid and had undergone a range of index surgical procedures; 15 patients had no surgical intervention during their index admission. The median Charlson Co‐morbidity Index score was 5·0 (0–10), and the median IMD decile was 4·0 (1–10), with 1 being the most deprived decile and 10 the least deprived. The median readmission LOS was significantly shorter than that of the index admission at 6·5 (0–78) days (*P* = 0·030).

**Table 1:** Demographic details of patients with an unplanned 30‐day vascular readmission

	Total 30‐day readmissions (*n* = 72)	Avoidable readmissions (*n* = 36)	Unavoidable readmissions (*n* = 36)	*P* ‡
**Age (years)** *	69·7 (19–93)	71·0 (31–93)	66·6 (19–93)	0·351§
**Sex ratio (M** : **F)**	49 : 23	21 : 15	28 : 8	0·071
**Co‐morbidity**				
PVD	63 (88)	33 (92)	30 (83)	0·252
Hypertension	48 (67%)	22 (61)	26 (72)	0·326
Diabetes mellitus	21 (29)	12 (33)	9 (25)	0·458
IHD	16 (22)	9 (25)	7 (19)	0·542
CKD	15 (21)	7 (19)	8 (22)	0·754
**Charlson Co‐morbidity Index score** *	5·0 (0–10)	5·0 (1–10)	5·0 (0–10)	0·622§
**IMD decile** *	4·0 (1–10)	3·5 (1–10)	4·0 (1–8)	0·890§
**Index LOS (days)** *	9·0 (1–155)	10·0 (1–155)	8·5 (2–141)	0·327§
**Readmission LOS (days)** *	6·5 (0–78)	8·0 (0–66)	5·0 (0–78)	0·050§
**Time to readmission (days)** *	10·0 (1–30)	8·5 (2–28)	12·0 (1–30)	0·450§
**Type of index admission**				0·588
Emergency	34 (47)	18 (50)	16 (44)	
Elective	25 (35)	10 (28)	15 (42)	
Urgent	13 (18)	8 (22)	5 (14)	
**Index surgical procedure**				0·118
Open AAA repair	7 (10)	2 (6)	5 (14)	
EVAR/TEVAR	7 (10)	2 (6)	5 (14)	
Lower‐limb bypass	8 (11)	3 (8)	5 (14)	
Lower‐limb endovascular repair	12 (17)	9 (25)	3 (8)	
Amputation	10 (14)	6 (17)	4 (11)	
Other†	13 (18)	4 (11)	9 (25)	
None	15 (21)	10 (28)	5 (14)	
**Discharge location**				0·878
Own home	57 (79)	28 (78)	29 (81)	
Home with social support	11 (15)	4 (11)	7 (19)	
Social care facility	4 (6)	4 (11)	0 (0)	

Values in parentheses are percentages unless indicated otherwise;

* values are median (range).

† For example carotid repair, deep venous reconstruction. PVD, peripheral vascular disease; IHD, ischaemic heart disease; CKD, chronic kidney disease; IMD, index of multiple deprivation; LOS, length of stay; AAA, abdominal aortic aneurysm; EVAR, endovascular aortic repair; TEVAR, thoracic endovascular aortic repair.

‡ Fisher's exact test, except §Mann–Whitney *U* test.

On average, patients readmitted within 30 days presented on day 10 (1–30) after discharge. The majority (56 of 72, 78 per cent) were readmitted via the hospital's emergency department, with 41 (57 per cent) readmitted directly under the care of vascular surgery team (*Table* 2). Readmissions were related predominantly to the index vascular procedure, with pain and wound complications accounting for 20 (28 per cent) and 26 (36 per cent) of the readmissions respectively. Other surgical complications accounted for a further four readmissions (6 per cent), and the remaining 22 (31 per cent) were admissions under medical specialties. A further surgical procedure was required in 21 readmitted patients (29 per cent).

**Table 2:** Details of 72 unplanned 30‐day readmissions

	Total 30‐day readmissions (*n* = 72)	Avoidable readmissions (*n* = 36)	Unavoidable readmissions (*n* = 36)	*P* *
**Route of readmission**				
Emergency department	56 (78)	29 (81)	27 (75)	0·822
Via outpatient clinic	13 (18)	6 (17)	7 (19)	0·822
Transferred	3 (4)	1 (3)	2 (6)	0·822
**Readmitting service**				
Vascular surgery	41 (57)	18 (50)	23 (64)	0·341
Other	31 (43)	18 (50)	13 (36)	0·341
**Reason for readmission**				
Pain	20 (28)	13 (36)	7 (19)	0·225
Wound complications	26 (36)	10 (28)	16 (44)	0·225
Other	4 (6)	1 (3)	3 (8)	0·225
Non‐surgical	22 (31)	12 (33)	10 (28)	0·225
**Surgical procedure during readmission**	21 (29)	11 (31)	10 (28)	0·802

* Fisher's exact test.

**Figure 2:**
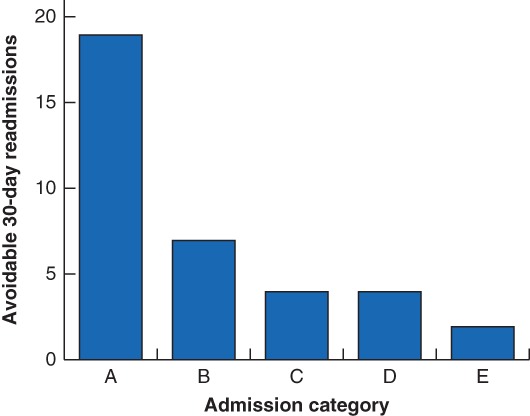
Bar chart showing subgroup classification by an expert panel of avoidable 30‐day readmissions in 36 patients Admission category: A, unresolved issue on discharge; B, inappropriate admission; C, inadequate social support; D, adverse event; E, other.

Following expert multidisciplinary peer review, 36 of the 72 unplanned 30‐day readmissions (50 per cent) were found to be potentially avoidable. *Fig*. 2 details the proportion of avoidable readmissions in each of the five categories. Nineteen (53 per cent) were due to an unresolved issue on discharge, such as the failure to treat a diabetic foot infection adequately during the index admission. This group of patients included a disproportionate number of the readmitted patients who required further surgical intervention: ten of the 11 secondary procedures required, the majority due to bleeding or infection‐related complications. Seven readmissions (19 per cent) were deemed inappropriate as there was potential for outpatient management of the admitting complaint, such as superficial wound infections that could be safely and effectively managed in the community. Four patients (11 per cent) were readmitted owing to inadequate social support; these were typically elderly or frail patients who were readmitted when their package of care in the community failed to support their initial discharge. A further four readmissions (11 per cent) were due to avoidable errors in management leading to adverse events, such as a patient admitted following drowsiness and a fall having been discharged on an incorrect dosage of opioid analgesia. The final two avoidable readmissions (6 per cent) could not be categorized by agreement of the panel; one was for episodes of recurrent hypertension that had initially been successfully treated before discharge, and the other was due to inappropriate presentation following the successful conservative management of a type B aortic dissection. There were no significant differences in the selected patient‐ and admission‐related factors between readmissions deemed to be avoidable and those that were not. Importantly, there were no significant differences in readmissions and their avoidability across the index procedure type or route of admission. Only an increased LOS after avoidable readmission was close to significance (median 8·0 days *versus* 5·0 days for unavoidable readmissions; *P* = 0·050) (*Table* 1).

## Discussion

Although avoidable readmissions in medical‐based specialties have been the subject of ongoing research for many years, avoidable readmissions in the surgical cohort have received far less attention^22^; just one study^14^ has sought to identify avoidable readmissions in surgery. This is surprising given the widespread use of surgical readmission rates as a marker of the quality of care^1^, ^23^ and source of financial penalties for hospitals. This study has identified and classified avoidable readmissions in vascular surgery, a specialty notoriously affected by high emergency 30‐day readmissions after inpatient care^17^.

In this single‐centre review of 885 discharges, 104 readmissions within 30 days of discharge were identified, of which approximately three‐quarters were unplanned. Half of unplanned 30‐day readmissions were found to be avoidable following expert multidisciplinary peer review, with failure to address ongoing medical issues adequately being the most common cause and no significant difference in patient factors found between the two groups. Defining the avoidability of readmission is a subjective process, but peer review by an expert panel is accepted as the objective standard.

The unplanned 30‐day readmission rate of 8·1 per cent is low compared with rates reported from other studies of 8·9–16·3 per cent^17^, ^24^, ^25^, ^26^, and the planned readmission rate of 26·9 per cent of total readmissions is close to the 25·3 per cent reported elsewhere^24^. Planned readmissions comprise a significant proportion of overall readmissions in vascular surgery, and should therefore be excluded when readmission rates are used to assess quality or guide the imposition of financial penalties on hospitals. The 50 per cent avoidable readmission rate reported in the present study is significantly higher than that in a similar study^14^ in general surgery, which found an avoidable readmission rate of just 21 per cent using a similar methodology across 258 readmissions. A further systematic review^22^ identified a median rate of 27·1 (range 5–79) per cent; however, this discrepancy should be interpreted with caution given the significant heterogeneity in defining and identifying avoidable readmissions in the studies evaluated.

Evaluating the preventability of readmissions after vascular surgery may provide valuable lessons that are likely applicable to all institutions. First, 19 of the 36 avoidable readmissions (53 per cent) were categorized as due to an unresolved issue on discharge, with a further seven (19 per cent) categorized as inappropriate admissions. This suggests that a subset of patients are being discharged having received inadequate care due to operational pressures or poor clinical decision‐making, or alternatively are not being provided with appropriate multidisciplinary team input and ongoing care following discharge to support their transition to community care successfully. In this vascular tertiary referral centre, the service is configured to provide consultant surgeon review within 12 h of admission, and a dedicated physician is also part of the multidisciplinary surgical team. The co‐management of surgical patients with ward‐based physicians has been shown to result in improved quality of care and lower readmission rates^27^, ^28^. In addition, improved coordination across the medical team and better clinical decision‐making have been linked to the rate of unplanned readmissions^29^.

Early discharge appears to be implicated in high readmission rates, but it has also been trialled successfully as a means of reducing LOS^30^, ^31^, albeit in an elective setting that excludes the challenges of managing patients with a vascular emergency. Analysis of more than 9000 patients undergoing thoracic aortic aneurysm and thoracic endovascular aortic repair procedures found that early discharge did not adversely impact 30‐day readmission rates or overall mortality^32^, and a fast‐track pathway for early discharge after complex aortic surgery has been shown to reduce LOS successfully without negatively affecting patient outcomes^31^. One of the lessons from reported success of early discharge plans might be that it is postdischarge follow‐up and multidisciplinary care during transition to the community that influences risk of readmission rather than the index LOS, or that expedited discharge is appropriate only for the elective pathway of care and is not safe or effective for patients admitted as an emergency.

A number of studies have examined the effect of postdischarge follow‐up on the risk of readmission in other complex patient cohorts, such as those with chronic obstructive pulmonary disease (COPD). In one such study^33^, patients with COPD who attended a follow‐up appointment within 30 days of discharge were 45 per cent less likely to be readmitted within 90 days. A similar study^34^ found that postdischarge follow‐up with either a primary care physician or respiratory physician did not alter risk of readmission or frequency of emergency department visits, but did result in a 62 per cent reduction in 30‐day mortality risk.

In the present study, four (11 per cent) of the 36 avoidable readmissions were due to inadequate social support after discharge. Various interventions that address the hospital‐to‐home transition, including home visits and telephone‐based follow‐up after discharge, have been proposed as a means of reducing unplanned readmissions, with varying success^35^. Two notable RCTs^36^, ^37^, however, were successful in reducing the risk of readmission in elderly patients through the use of nurse‐led discharge planning and home care interventions. Intensive postdischarge follow‐up, combining both patient and caregiver education, reconciliation of medication and regular reminders to ensure attendance at follow‐up appointments, has also been shown to be successful^38^. By addressing multiple areas of potential shortfall within the transition of care, it is possible to reduce unplanned readmissions through a multifactorial approach to postdischarge support, rather than through interventions that target discrete areas of the transition from secondary to community care.

There are limitations to this study that must be considered. First, there is an inherent selection bias associated with a retrospective chart review that could not have been avoided in this study design. Second, as full data collection was limited to the readmitted cohort, it was not possible to comment on factors predicting risk of readmission; thorough interrogation of data for patients who were not readmitted would have allowed for this. An additional limitation was the 28·6 per cent exclusion rate from the original 1239 consecutive patients identified, with almost half of these being due to coding errors and duplicate data. Patients are advised always to return to the hospital where their primary procedure was done, and the records reviewed covered admissions to the five constituent hospitals that form the organization. It is likely, however, that some admissions to other hospitals occurred. Standardized methodology for assessing preventability of readmission would aid comparison across centres.

With a median length of stay of 8·0 days for the potentially avoidable readmissions and an average cost per bed‐day in the NHS of around £250 (€280, exchange rate 21 June 2019)^39^, preventing these readmissions would mean a potential saving of £2000 (€2237) per readmission, a potential saving of more than £6·5 million (€7·3 million) per year for the 43 000 vascular procedures performed each year in England. This conservative estimate represents solely the cost of the hospital bed and does not account for cost of treatment, drugs, further interventions, additional resources required or associated financial penalties. Further analysis of the true cost of these avoidable readmissions is therefore required to evaluate fully the total financial outlay and resource implications, and to provide added impetus to effect positive change.

Efforts should be geared towards optimizing the discharge process for patients undergoing vascular surgery, supporting the transition from hospital to community care through a multidisciplinary approach to care and implementing strategies targeted at those patients deemed to be at high risk of readmission in order to tackle this important aspect of care quality in the high‐need, high‐cost cohort of patients who undergo vascular surgery.
